# The Applications of Ferulic-Acid-Loaded Fibrous Films for Fruit Preservation

**DOI:** 10.3390/polym14224947

**Published:** 2022-11-16

**Authors:** Xinyi Huang, Wenlai Jiang, Jianfeng Zhou, Deng-Guang Yu, Hui Liu

**Affiliations:** 1 School of Materials and Chemistry, University of Shanghai for Science and Technology, Shanghai 200093, China; 2 Shanghai Engineering Technology Research Center for High-Performance Medical Device Materials, Shanghai 200093, China

**Keywords:** polyethylene oxide, ferulic acid, maize protein, electrospinning, antioxidant, food packaging

## Abstract

The aim of this study was to develop a novel ultrathin fibrous membrane with a core–sheath structure as an antioxidant food packaging membrane. The core–sheath structure was prepared by coaxial electrospinning, and the release of active substances was regulated by its special structure. Ferulic acid (FA) was incorporated into the electrospun zein/polyethylene oxide ultrathin fibers to ensure their synergistic antioxidant properties. We found that the prepared ultrathin fibers had a good morphology and smooth surface. The internal structure of the fibers was stable, and the three materials that we used were compatible. For the different loading positions, it was observed that the core layer ferulic-acid-loaded fibers had a sustained action, while the sheath layer ferulic-acid-loaded fibers had a pre-burst action. Finally, apples were selected for packaging using fibrous membranes to simulate practical applications. The fibrous membrane was effective in reducing water loss and apple quality loss, as well as extending the shelf life. According to these experiments, the FA-loaded zein/PEO coaxial electrospinning fiber can be used as antioxidant food packaging and will also undergo more improvements in the future.

## 1. Introduction

Food is the essential source of energy for human production and daily life, and its research and development, production, and transport across the whole industry chain are closely related to food packaging. Food packaging works in three ways: firstly, to ensure that the food is not contaminated during transportation; secondly, to ensure the overall freshness of the food itself; and thirdly, to provide consumers with portability and convenience [1]. With the fast pace of modern life, people’s demand for prepared food has changed. As a result, the upgrading of food packaging and the addition of food components have become the most intuitive ways to improve food storage life [2,3]. The principles of shelf-life extension are threefold: reducing the rate of oxidation, inhibiting the growth of food bacteria, and slowing down the process of autonomous respiration [4,5,6]. Most traditional food preservation technologies on the market are based on increasing cold chain transport and setting up low-temperature vending areas to extend the food cycle. Although this method of extending the shelf life of food by lowering the temperature can be effective, it fails to achieve the desired effect. The overall freshness is greatly reduced by the constant attack of bacteria and free radicals from outside [7].

Intuitive ways to prolong the shelf-life of food products include the addition of preservatives in food production and improvements in food packaging. The former is controversial due to dosage standards and safety issues with preservatives. This has led to the development of new packaging technologies that incorporate active substances into food packaging, i.e., active food packaging, which uses active ingredients to react with a range of substances to preserve the freshness of food [8].

However, the use of functional active substances must meet food safety standards, so that the active substance can perform its specific function under safe and ingestible conditions [9]. The focus of attention is on natural substances derived from plants, including essential oils and polyphenols [10,11,12]. Due to their specific chemical structure, the phenolic substances they contain can react with peroxyl radicals [13].

As a result, essential oils and phenolics have become popular in research. Although natural essential oils and phenols have the advantage of being non-toxic and safe, they are unstable and volatile. Therefore, traditional food packaging processing methods can easily affect their effectiveness and even lead to overall failure. Electrospinning technology, with its simplicity and room temperature manipulability, makes it possible to encapsulate natural functional substances into food packaging in the production chain [14,15]. 

Electrospinning is a top-down technique in which polymer solutions are stretched into micro- and nanofibers by electrostatic field forces [16,17,18,19,20,21], often with room temperature operability. It is an easy method for transforming filament-forming polymers into nanofibers [22,23,24,25] and has become one of the most powerful tools in the polymeric engineering field [26,27,28]. Although electrospun single-component polymer nanofibers can be exploited directly [29], in most cases the functional ingredients are co-dissolved with a polymer to form a blended fluid [30,31,32,33,34]. After solidification by electrospinning, the polymeric nanofibers are endowed with a certain functional application [35,36,37]. Many new encapsulation methods were invented alongside the development of electrospinning, such as coaxial [38,39] and side-by-side electrospinning [40], as well as the electrospinning of nano suspensions contained in drug-loaded nanoparticles [41].

In 2003, Sun et al. [42] reported a core–sheath structure for coaxial electrospinning, allowing the polymer to be sequestered into the nanofibers. Coaxial electrostatic spinning fiber technology has made it possible to modulate the structure to control the rate of release of active substances [43,44,45,46,47,48]. The overall technological process is suitable for the expected release of functional actives from food packaging. Electrospinning is quickly moving forward to tri-axial, modified tri-axial, side-by-side, and tri-fluid Janus processes [49,50,51,52,53,54]. Coaxial electrospinning is one of the most popular processes due to its relative simplicity in implementation, in addition to the usefulness of double-layer core–shell structures [55,56,57]. Therefore, coaxial electrospinning technology was adopted for this experiment to develop antioxidant food active packaging [58].

Zein (from corn), used in this experiment, is an alcohol-soluble protein derived from natural food corn with high thermal stability and biocompatibility [59]. It is considered promising for use as a conveyor belt for the delivery of functional active substances. The combination of zein and polyethylene oxide (PEO), a water-soluble polymer with good compatibility and degradability, can enhance the overall solution’s textile and mechanical properties [60,61].

Ferulic acid (FA), a phenolic acid with antioxidant properties, is found abundantly in plants and vegetables [62,63,64]. FA itself has been shown to have outstanding anti-aging and anti-cancer properties in pharmacology studies. Due to the reliability demonstrated by the FDA as well as its stability under UV light [65], FA was used as the functional substance in this experiment. Niloufar Sharif et al. [66] illustrated the spinnability of ferulic acid by encapsulating it in hydroxypropyl-beta-cyclodextrin and successfully preparing electrospinning fibers.

Combining previous studies and the properties of various substances, we chose apple slices as the experimental object, aiming to investigate the coaxial core–sheath electrospinning functional nanofibers loaded with FA, using zein and PEO as the electrospinning polymer matrix, to achieve the antioxidant target. The composition and morphology of the fibers were characterized, and the antioxidant effect was tested.

## 2. Experiments

### 2.1. Materials

The chemical substances, which included ethanol, polyethylene oxide (PEO), and ferulic acid (FA), were obtained from Sinopharm Chemical Reagent Co., Ltd. (Shanghai, China). Zein (from corn) was purchased from Tokyo Chemical Industry Co., Ltd. (Tokyo, Japan). All chemicals were of analytical grade, and water was doubly distilled through a purification system.

### 2.2. Electrospinning Preparation

**Figure 1:**
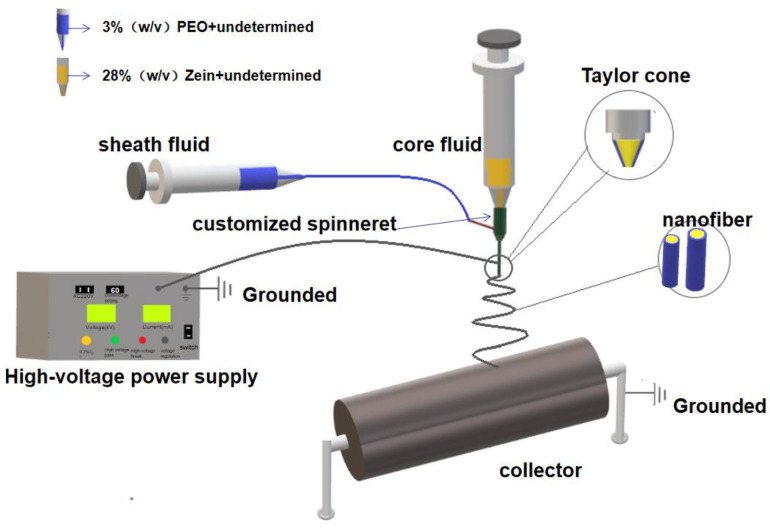
Schematic diagram of the coaxial electrospinning system.

**Figure 2:**
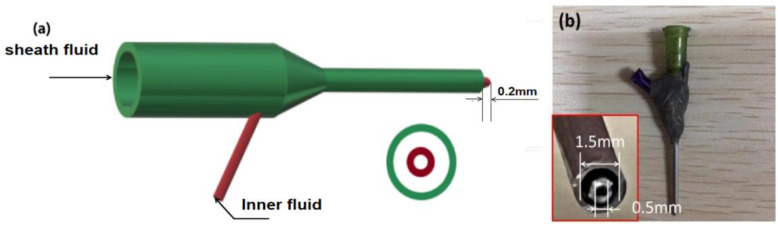
Structure of the homemade coaxial spinneret: (**a**) Diagram of the designed structure of the coaxial spinneret; (**b**) coaxial spinneret and the head of the spinneret (bottom-left insert).

This experiment was performed using a customized electrospinning system, as shown in Figure 1 and Figure 2 below. The spinning equipment consisted of high voltage (60 kV/2 mA) power (ZGF2000, Shanghai Suter, China), two syringe pumps (KDS100 and KDS200, Cole Palmer, IL, USA), a customized coaxial spinning nozzle, and aluminum foil as a collector.

The experimental group consisted of two fluids as the core and sheath solutions for coaxial electrospinning. For the core solution, 3% (*w/v*) PEO solution was prepared by adding 3 g PEO to 100 mL of 80% *v/v* ethanol aqueous solution and stirring with a magnetic stirrer for 8 h. For the sheath solution, 28% (*w/v*) zein solution was prepared by dissolving 28 g zein in 100 mL of 80% *v/v* ethanol aqueous solution and stirring with a magnetic stirrer for 8 h. Adjusting the content of ferulic acid in the core or sheath solution allowed us to set up control groups. The detailed parameters are shown in Table 1 below.

The electrostatic spinning process parameters were progressively optimized through a series of pre-experiments, including uniaxial and coaxial electrostatic spinning to prepare zein–PEO fibers. The experimental parameters were a high-voltage power supply set at 7.0 kV; a collection distance of 20 ± 2 cm; a core fluid flow rate of 0.6 mL/h; a sheath fluid flow rate of 0.3 mL/h; an ambient temperature setting of 25 ± 2 °C; and an experimental relative humidity of 60 ± 5%.

### 2.3. Characterization of the Experimentally Obtained Fiber Components

The experimentally obtained fiber components were characterized for various properties. For the Quanta FEG 450 scanning electron microscope (SEM, FEI Co., Ltd., Hillsboro, OR, USA), we used vacuum evaporation equipment to place the sample on thin layers of gold. The internal groups of the electrospinning fibers or raw materials were analyzed using attenuated total reflection Fourier transform infrared spectroscopy (ATR-FTIR, Spectrum 100). The scan range was set to 450 to 4000 cm^−1^, and the number of scans was 8. The physical state of the prepared nanofibers and raw materials was analyzed using X-ray diffraction (XRD). The angle of incidence 2θ was set in the range of 5–60°, the voltage was 40 kV, and the current was 30 mA.

### 2.4. FA Releasability

In order to evaluate the FA release behavior of the nanofibers when exposed to foods with a high moisture content, the 24 h dissolution concentration profile of FA was studied using an SHZ-86 water bath thermostatic oscillator.

A conical flask was filled with 125 mL of deionized water and immersed in a water bath shaker with the temperature set at 25 ± 0.5 °C and the speed fixed at 50 rpm. The FA dissolution test was started when the temperature of the deionized water in the conical flask and the temperature of the water bath were the same. The FA-loaded fiber membrane was cut into rectangles weighing 25 ± 0.5 mg and placed in the conical flask. The moment that the membrane was placed in the conical flask was recorded as the zero moment, and 2.5 mL of solution was removed from the conical flask using a pipette to measure the FA concentration at equal intervals. At the same time, 2.5 mL of distilled water was removed from the spare conical flask and added to the conical flask from which the sample solution had been removed to maintain a constant total volume of solution in the conical flask.

The concentration of FA (F%) in the test solution was calculated using the following Equation (1):

(1) $$F\%=\frac{X_{n}\times V_{0}+\sum_{i=1}^{n-1}X_{i}\times V}{Q_{0}}\times 100\%$$

*X_n_* represents the concentration of the nth sample solution taken, *X_i_* represents the concentration of the *i*-th sample solution taken, *V* represents the volume of solution removed each time, 2.5 mL, and *V*_0_ represents the total volume of solution in the conical flask.

### 2.5. Analysis of Antioxidant Properties

The antioxidant properties of the preparations were analyzed using the DPPH radical scavenging method [67,68,69,70,71,72]. We prepared a DPPH standard solution at a concentration of 60 mg/L and placed 10 mg of the functional nanofiber membrane in 10 mL of anhydrous ethanol. A total of 0.1 mL of the sample solution and 2.5 mL of DPPH standard solution were collected and shaken well in a centrifuge tube for 5 min, 1 h, and 24 h. The absorbance of the DPPH solution after this reaction was measured at a wavelength of 516 nm, noted as A_sample_, and the absorbance of the DPPH standard solution before the chemical reaction was carried out, noted as A_background_, and the reduction in absorbance of the DPPH solution was used to determine the antioxidant activity of the reaction fiber (AA%). Equation (2) is shown below.

(2) $$\mathrm{AA}\%=\frac{A_{\mathrm{background}}-A_{\mathrm{sample}}}{A_{\mathrm{background}}}\times 100\%$$

### 2.6. Fruit Preservation

Equal slices of purchased fresh apples were packed in direct contact with the PEO/zein nanofiber membranes. The apples were divided into five groups; the first group was a blank control group, and the other four groups were packed using F4, F5, F6, and F7 fibers. The mass of the apple slices was weighed separately at each pre-determined time point to calculate the weight loss rate to analyze the degree of freshness retention of the fiber membrane.

Equation (3) for calculating the weight loss rate is as follows:

(3) $$\mathrm{weight}\;\mathrm{loss}\;\mathrm{rate}\left(\%\right)=\frac{W_{0}-W_{i}}{W_{0}}\times 100\%$$

where W_0_ is the initial mass of the apple slice and W_i_ is the mass of the apple slice at the i-th observation time.

Equal slices of purchased fresh apples were placed in beakers and packaged with the aid of PEO/zein nanofiber membranes. The nanofiber membrane was cut into 30 mg pieces, and the packaging process lasted for 8 days at an experimental temperature of 4 ± 1 °C. The apples were divided into five groups; the first group was a blank control group, and the other four groups were packed using F4, F5, F6, and F7 fibers. The content of benzoquinone, a browning product of apple slices, was measured at each preset time point to analyze the browning rate.

### 2.7. Statistical Analysis

The data were statistically analyzed using Microsoft Windows Excel 2019 and OriginLab Origin 2021. Data were expressed as the means ± standard deviations (SD). All experiments were conducted in triplicate.

## 3. Results and Discussion

### 3.1. Coaxial Electrospinning

The compositions of a coaxial system have no essential differences to a traditional single-fluid process or monoaxial process. Shown in Figure 1 is a diagram of a coaxial system. The only difference is that two working fluids are simultaneously led to a concentric spinneret. The spinneret is the most important part in an electrospinning system. It determines the types of electrospinning processes, the final structure of the resultant nanofibers, and even the production yields [73].

In our study, a homemade spinneret was designed; a diagram and digital picture are included in Figure 2. The characteristics of this concentric spinneret include the following: (1) the core capillary inserts into the shell capillary from an enlarged section of one side (Figure 2a); (2) the spinneret consists of both polymers and stainless steel (Figure 2b), which is useful for preventing energy loss to the environment [74] regardless of AC or DC power supply [75]; (3) the top of the inner capillary slightly projects out of the surface of the shell capillary, which is useful for a better encapsulation of the core fluid by the shell liquid. The concentric spinneret had an inner and outer diameter of 0.5 and 1.5 mm, respectively (Figure 2b).

### 3.2. Uniaxial Electrospinning Fiber Morphology

**Figure 3:**
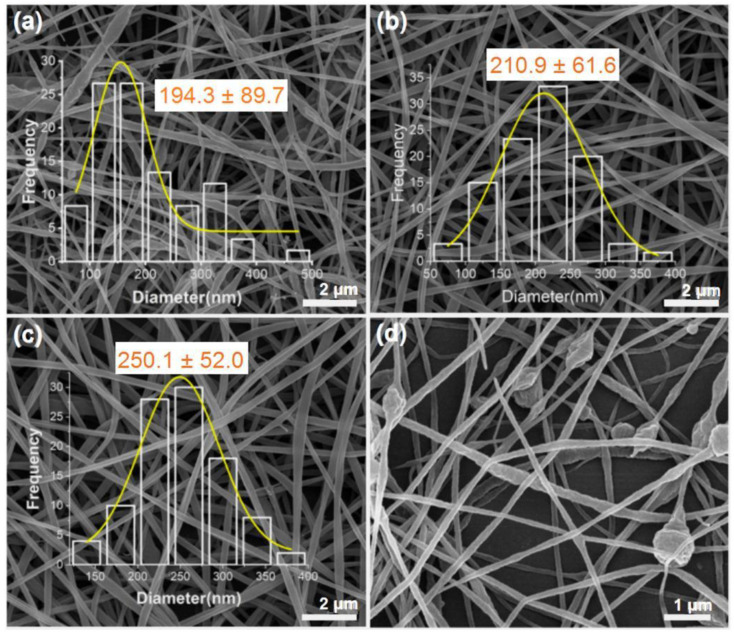
SEM images of zein fibers with different ferulic acid loadings: (**a**) 0% FA; (**b**) 10% FA; (**c**) 15% FA; (**d**) 20% FA.

The optimum concentration of the zein solution for spinning was 28%, so all subsequent experiments were carried out based on this parameter. The experiments were carried out by setting four control groups, with 0%, 10%, 15%, and 20% ferulic acid loadings for the polymer matrix zein. The SEM images obtained are shown in Figure 3 below: when the zein was not loaded with ferulic acid, the nanofibers obtained had an uneven distribution of diameters and large fluctuations in the values of individual fiber diameters, whereas when loaded with ferulic acid, the fibers had a flatter shape and a more uniform diameter distribution. The overall shape of the fibers increased with increasing ferulic acid loading, but at 20% ferulic acid loading, the fibers exhibited a beaded morphology.

As a high ferulic acid loading can lead to the agglomeration of the overall textile fiber, the ferulic acid loading can alter the overall flow of the zein solution. The images demonstrated that the fibers were not homogeneously arranged after 20% ferulic acid loading; therefore, the optimum concentrations of 10% and 15% ferulic acid were chosen for the subsequent coaxial electrostatic spinning preparation.

### 3.3. Coaxial Electrospinning Fiber Morphology

**Figure 4:**
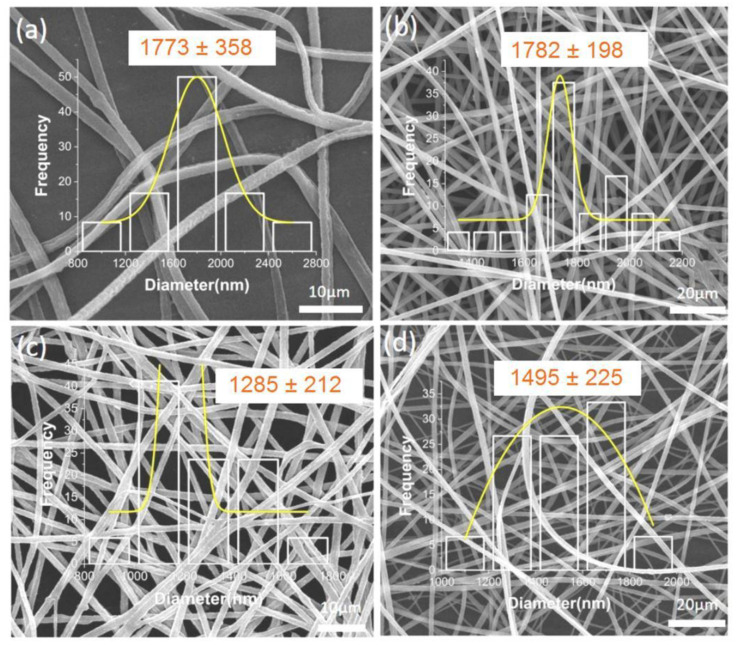
SEM image of coaxial electrospinning fibers loaded with FA: (**a**) F4 fiber loaded with 10% FA in the core layer; (**b**) F5 fiber loaded with 10% FA in the sheath layer; (**c**) F6 fiber loaded with 15% FA in the core; (**d**) F7 fiber loaded with 15% FA in the sheath layer.

The prepared F4 to F7 coaxial electrospinning fibers were characterized as shown in Figure 4a,c for the coaxial core layer ferulic-acid-loaded fibers and as shown in Figure 4b,d for the coaxial sheath layer ferulic-acid-loaded fibers. The overall morphologies of the sheath-loaded fibers indicated a larger diameter, and the surface of the fibers was flatter and smoother than that of the core-loaded fibers for the same parameter values. The reason for this change in diameter was that when the sheath layer was loaded with ferulic acid, the zein structure of the sheath layer solution expanded in volume and therefore increased in diameter. When the core layer was loaded with ferulic acid, the zein in the sheath structure wrapped around it, preventing a large change in the overall volume. The decrease in the coaxial fiber diameter with increasing ferulic acid loading also indicated that the effect of increasing the amount of active substance on the volume during the spinning process was less than the effect of the active substance on the properties of the spinning solution. The increased ferulic acid content improved the fluid properties of the two layers of the coaxial electrospinning core sheath, leading to a possible increase in solution viscosity or an increase in solution conductivity. These changes led to a finer stretching of the fluid at the tip of the Taylor cone, resulting in a smaller fiber diameter.

Overall, the four groups of coaxial electrospinning fibers had a relatively homogeneous distribution of diameters and a good overall morphology. This fiber structure facilitated the subsequent release of ferulic acid and provided a good experimental basis for the subsequent preparation of antioxidant active packaging.

### 3.4. Comparison of Two Electrospinning Fibers

**Figure 5:**
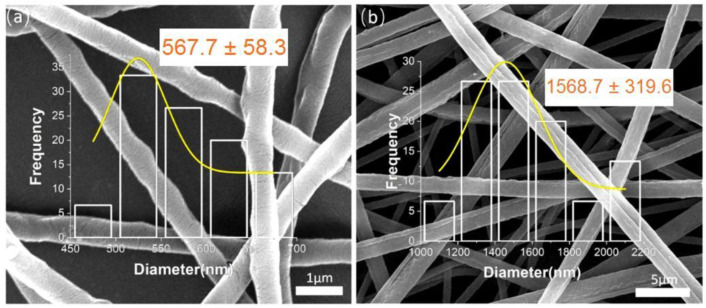
Comparison of uniaxial and coaxial electrospinning fibers: (**a**) uniaxial PEO fiber; (**b**) coaxial PEO–zein fiber.

The morphological comparison between the uniaxial nanofibers and coaxial nanofibers is shown in Figure 5a which corresponds to the observation of the uniaxial PEO fiber at 40,000 magnification, and Figure 5b which corresponds to the observation of the coaxial PEO–zein fiber of at 10,000 magnification. According to the analysis in the previous subsection, the uniaxial PEO fibers were unevenly coarse and thin, and the surface was not smooth in a curved and creeping manner, which showed from the side that the uniaxial electrospinning process of PEO was more unstable. The coaxial electrospinning fibers had a uniform distribution of diameters and showed a large increase in diameter compared to the uniaxial fibers. As a result, coaxial electrospinning fibers had a larger fiber surface area and were more absorbent than the uniaxial electrospinning fibers. This showed that the coaxial fibers will be more conducive to the release of active substances in functional food packaging.

### 3.5. Analysis of the Internal Components of the Nanofibers

**Figure 6:**
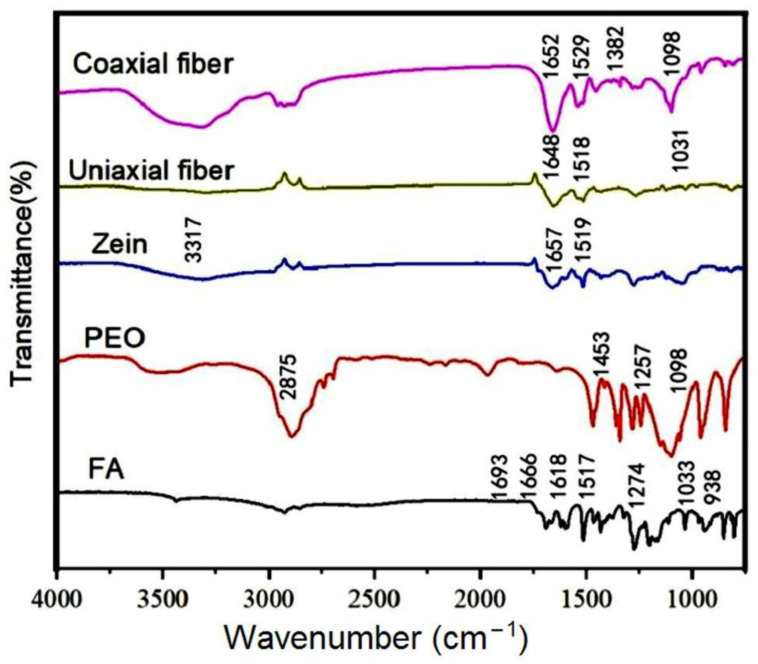
FT-IR spectra of raw materials and electrospinning fibers.

The internal fractions were determined by using Fourier transform infrared spectroscopy. The changes in the internal functional groups of the material before and after preparation were confirmed and the fractions characterized, as Figure 6 shows.

FA had a large number of characteristic peaks at 1750 to 750 cm^−1^, which included carbonyl vibrations, carbon–carbon double bonds, carbon–carbon single bonds, hydrocarbon bonds, and carbon–oxygen single bonds. These bonds also had different vibrational forms reflecting the ordered crystal structure of the FA molecule; characteristic peaks at 1657 and 1519 cm^−1^ were found in zein polymers, which corresponded to the vibrations of the carbonyl group and the amino group, respectively. Characteristic peaks at 2875, 1453, 1257, and 1098 cm^−1^ were found in PEO, representing the presence of the methyl and carbonyl groups in PEO.

**Figure 7:**
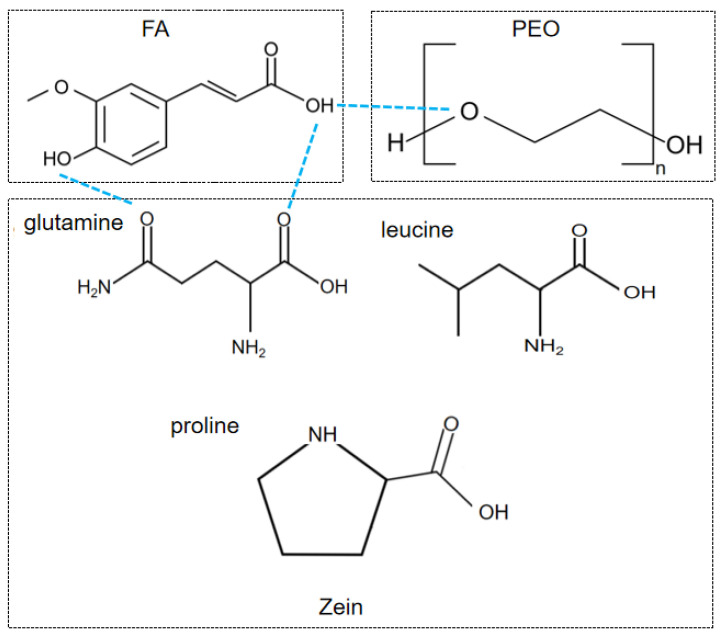
The molecular formulas of zein, PEO, and FA.

In the IR diffraction of the uniaxial and coaxial fibers, the characteristic peaks of FA disappeared despite the presence of the FA active substance. The FA molecules were distributed as solid dispersions in the nanofibers instead of crystals and showed good compatibility with the polymer matrix in a more stable bonding. At the same time, the peaks at 1657 and 1519 cm^−1^ from pure zein shifted slightly to the left or right in the fibers, presumably resulting in hydrogen bonding between FA and the polymer matrix, as shown in Figure 7, which illustrated a good and stable loading of FA in the fibers and facilitated the controlled release of the FA active substance.

### 3.6. XRD Analysis of Raw Materials and Electrospinning Fibers

**Figure 8:**
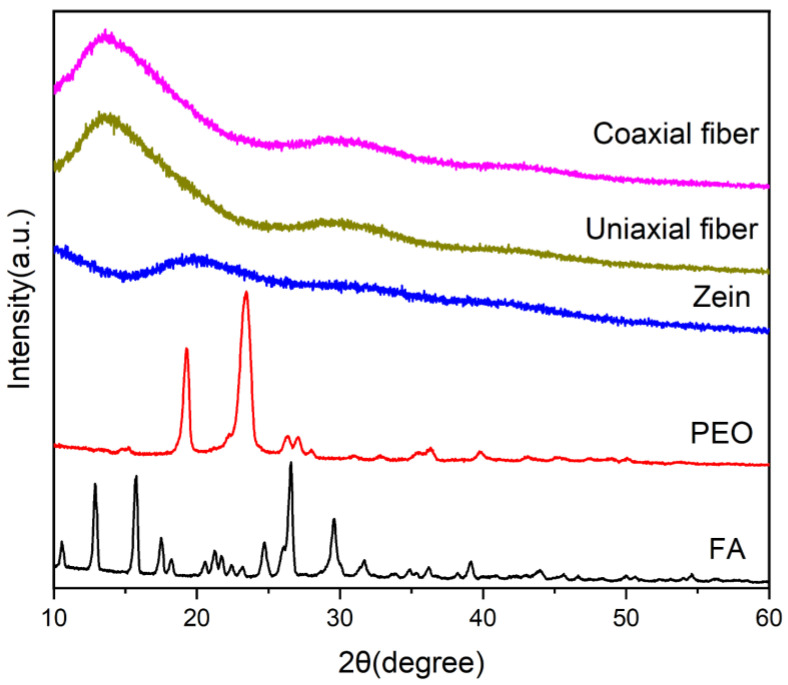
X-ray diffraction analysis of raw materials and electrospinning fibers.

XRD is a well-known technique used to characterize crystalline structures [76,77,78,79]. In this study, we used it to characterize the three raw materials, PEO, zein, and FA, as well as the uniaxial and coaxial fibers. The characterization results obtained are shown in Figure 8.

A comparison of the three figures showed the presence of several sharp peaks in the active substance FA, which indicated the presence of a crystalline form. As revealed by the several sharp peaks in the PEO polymer, PEO had a certain degree of crystallinity. The absence of sharp peaks in the zein polymer represented its amorphous physical form.

The observation of the uniaxial and coaxial fibers showed that the overall amorphous form of the fiber was present. Therefore, it was assumed that the FA actives had lost their original crystalline structure in the fibers and were only embedded as individual molecules in the resulting nanofiber polymer chains. The active substance was loaded homogeneously in the polymer, as revealed by this circumstance. This phenomenon satisfied the concept of the functional fiber packaging and the controlled release properties of the active substance.

### 3.7. Preparation of Standard Curves for FA

The standard absorbance/concentration curves of FA were prepared using a UV–Vis spectrophotometer to measure the absorbance of ferulic acid at different concentrations. The standard solution of 50 μg/mL of FA was used as the starting point for the preparation of FA solutions diluted 2-fold, 3-fold, 4-fold, 5-fold, 8-fold, and 10-fold.

**Figure 9:**
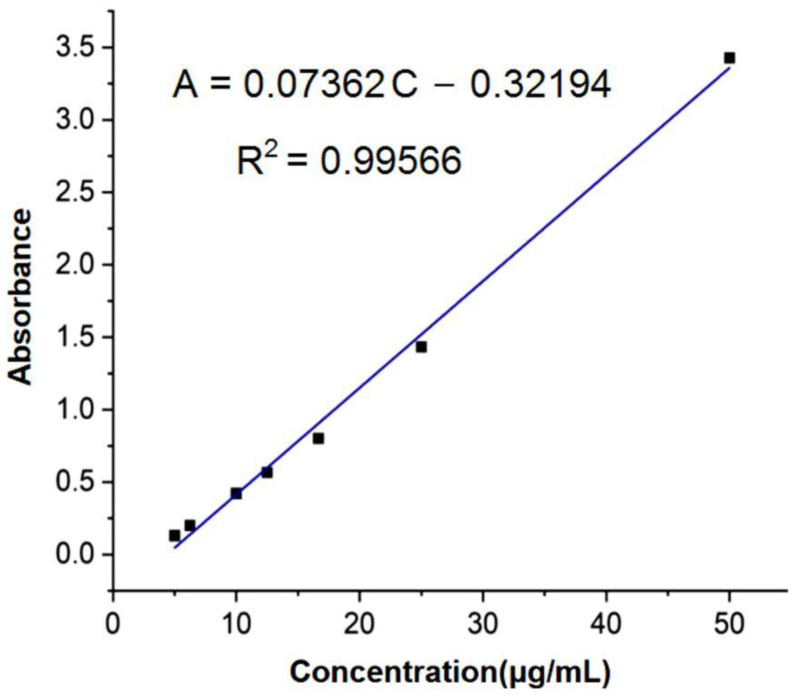
The standard curve of FA.

Based on the literature review, 322 nm was determined as the maximum absorption wavelength of FA, and the absorbance of each concentration of the solution was tested at this wavelength. As shown in Figure 9, a linear fit resulted in the primary linear Equation (4):

(4) $$A=0.07362C-0.32194\;\left(R^{2}=0.99566\right)$$

where A is the absorbance and C is the concentration of the FA solution. The fitting equation was used as the basis for the subsequent FA release experiment.

### 3.8. Analysis of FA Release Characteristics

**Figure 10:**
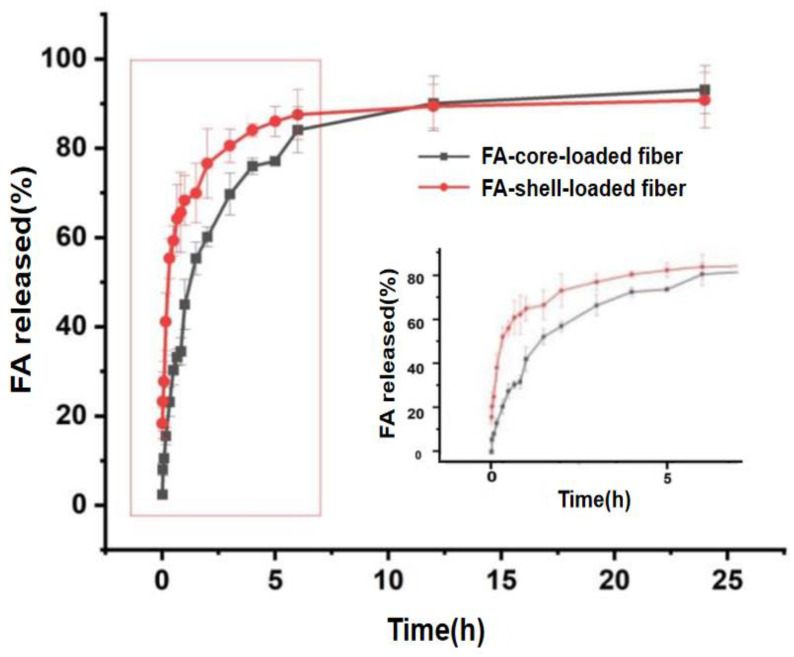
Analysis of the dissolution curve of FA and its local magnification.

The experimental temperature was controlled at 25 °C using a water bath thermostatic remote bed to simulate the process of packaging food products with a high moisture content and to analyze the dissolution characteristics of FA in distilled water in the F6 and F7 fibers. The dissolution curves obtained are shown in Figure 10 below.

The core layer-loaded fiber F6 and the sheath layer-loaded fiber F7 had a more pronounced release variability during the first five hours. In the first minute of the dissolution experiment, the FA release of the F6 fiber was 2.40%, compared to 18.33% for the F7 fiber. This indicated that the core layer carrier fibers had good burst release arresting properties and that the ferulic acid encapsulated in the sheath surface was able to precipitate rapidly under the influence of water molecules, as it was in direct contact with water on the fiber surface. A comparison of the overall time to 60% ferulic acid release showed that the core layer F6 fiber lasted 1.5 h longer than the sheath layer F7 fiber, demonstrating the controlled release of the core-layer structure.

As both fibers have their own characteristics, they have distinct potential applications in food packaging. The core layer carrier fiber F6 is more suitable for food packaging where sustained effectiveness is required due to its sustained action. The sheath layer carrier fiber F7, on the other hand, is more suitable for food packaging that is highly susceptible to rapid oxidation due to its good initial burst.

### 3.9. Analysis of the Antioxidant Properties of Nanofibers

The antioxidant activity of the fibers was reflected using the DPPH radical scavenging rate. The antioxidant activity of fibers F4, F5, F6, and F7 was studied separately, using the solutions of the fibers after dissolution in ethanol as the test sample and adding them to the DPPH radical solution to test their radical scavenging ability.

**Figure 11:**
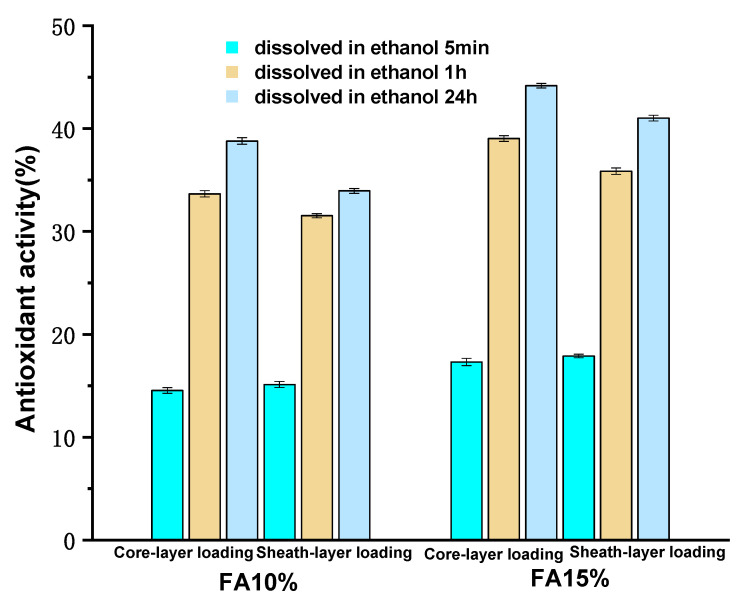
Analysis of the antioxidant activity tests on the fibers.

Figure 11 shows that the antioxidant activity of the fibers in ethanol gradually increased with time. The growth rate of the antioxidant activity of the fibers was faster from 5 min to 1 h and reached the maximum antioxidant activity at 24 h. In a comparison of the antioxidant activity of F4 to F7 when dissolved in ethanol for 24 h, it was observed that the antioxidant activity of the fibers increased when the loading of ferulic acid increased for the same dissolution time, which proved that a good loading of FA has antioxidant properties.

When comparing the core-loaded fibers with the sheath-loaded fibers, we found that the antioxidant activity of the sheath-loaded FA fibers was greater than that of the core-loaded FA fibers at 5 min of dissolution, while the antioxidant activity of the core-loaded FA fibers was stronger after 1 h and 24 h of dissolution. This confirmed the theory of the FA release experiment in 3.8, suggesting that the sheath-loaded FA fibers would dominate the efficacy in the early stages, while the core-loaded FA fibers would have a greater capacity for long-lasting effects.

### 3.10. Contact Fruit Packaging

**Figure 12:**
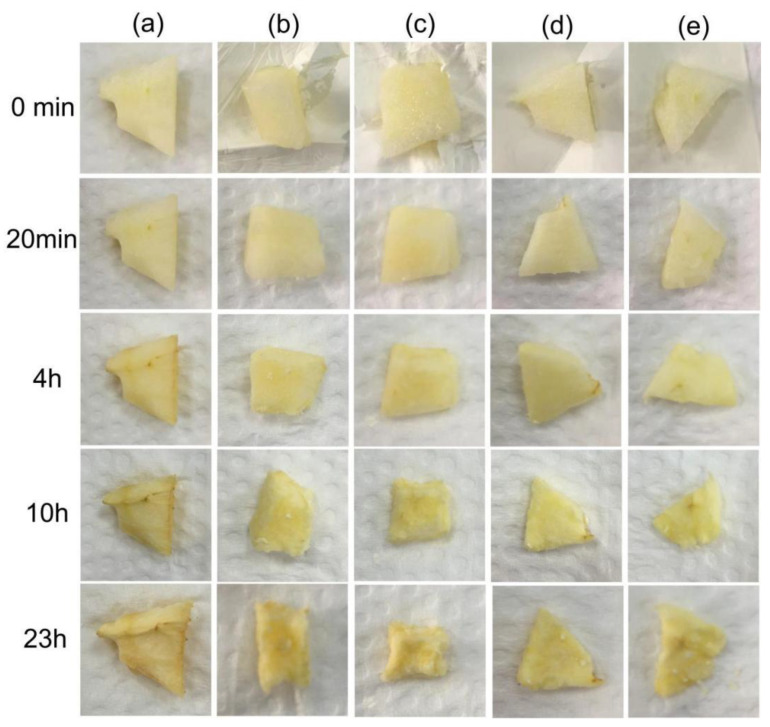
Contact packaging of apple slices and their appearance: (**a**) No fiber packaging; (**b**) F4 packaged group; (**c**) F5 packaged group; (**d**) F6 packaged group; (**e**) F7 packaged group.

The apple slices were packed directly in the fiber membrane at room temperature, and process changes were recorded by observation at a preset time, as shown in Figure 12 below. Groups (a) to (e) were the blank control and the apple slices packed with fibers F4 to F7, respectively.

No significant changes were observed in all experimental groups between the 0 min and 20 min stages. At 4 h, the apple slices in the blank group in (a) showed significant oxidation, with a dull yellow color and light yellow-brown edges. The experimental group with the fiber wrap did not show any significant changes, so it was clear that the prepared nanofiber wrap was feasible for antioxidation applications. When the overall experiment was carried out at 10 h, the oxidative browning of the apple slices in the blank group (a) increased, while no significant browning occurred in groups (b) to (e) where the nanofiber wrapping was used, demonstrating the antioxidant sustainability of the membranes. A parallel comparison of the four groups with different fiber wraps, F4 and F6, showed better preservation of the apple slices, confirming the stronger effect of the core-loaded FA fibers in terms of long-term antioxidant activity. At 23 h, all subjects had lost their food value.

### 3.11. The Weight Loss Rate of Contact Fruit Packaging

**Figure 13:**
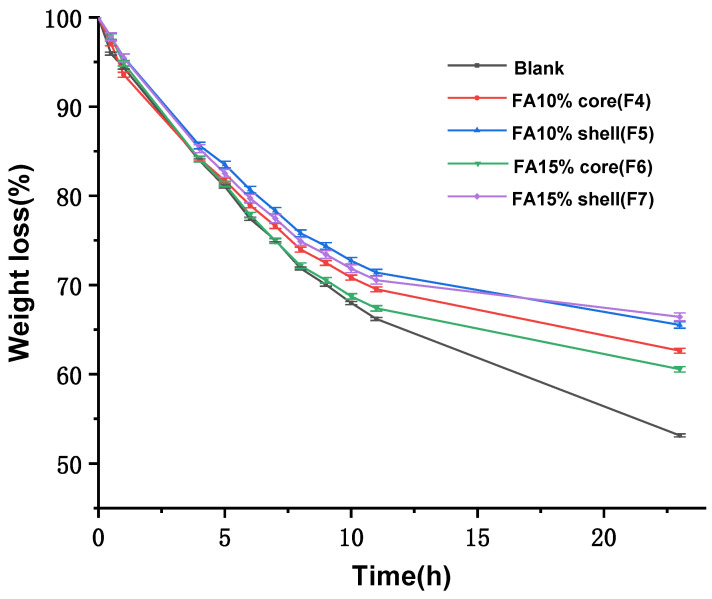
Weight loss analysis of apple slices after contact packaging.

In order to further analyze the degree of water loss and the ability of the fiber membrane to retain freshness, a quality test of the apple slices was carried out. The weight loss rate of the apple slices is shown in Figure 13.

In comparing the blank group with the fiber group, the quality of the blank group decreased to 53.14% by 23 h, while the apple slices packed in F4 to F7 decreased to 62.63%, 65.52%, 60.56%, and 66.43%, respectively. The overall mass loss and the water loss rate of the apple slices decreased after the use of fiber packaging. It can therefore be stated that the prepared nanofibers mitigated the water loss to a certain extent, and the F5 and F7 fibers showed better experimental results than the F4 and F6 fibers. The presence of both hydrophilic and hydrophobic amino acids within the maize alcohol protein, with the majority of hydrophobic amino acids causing the fibers to absorb water but not completely dissolve the fiber structure, is indicative of the water loss phenomenon.

The experiment therefore showed that the fibers absorbed water in direct packaging, and their enhanced hydrophobicity slowed the loss of water from the apple slices when they had absorbed sufficient water. The smooth fibers F5 and F7 had a smaller specific surface area than the rougher fibers F4 and F6, making them less likely to absorb water and thus helping to preserve the freshness of the apples.

### 3.12. Ancillary Fruit Packaging Morphology

**Figure 14:**
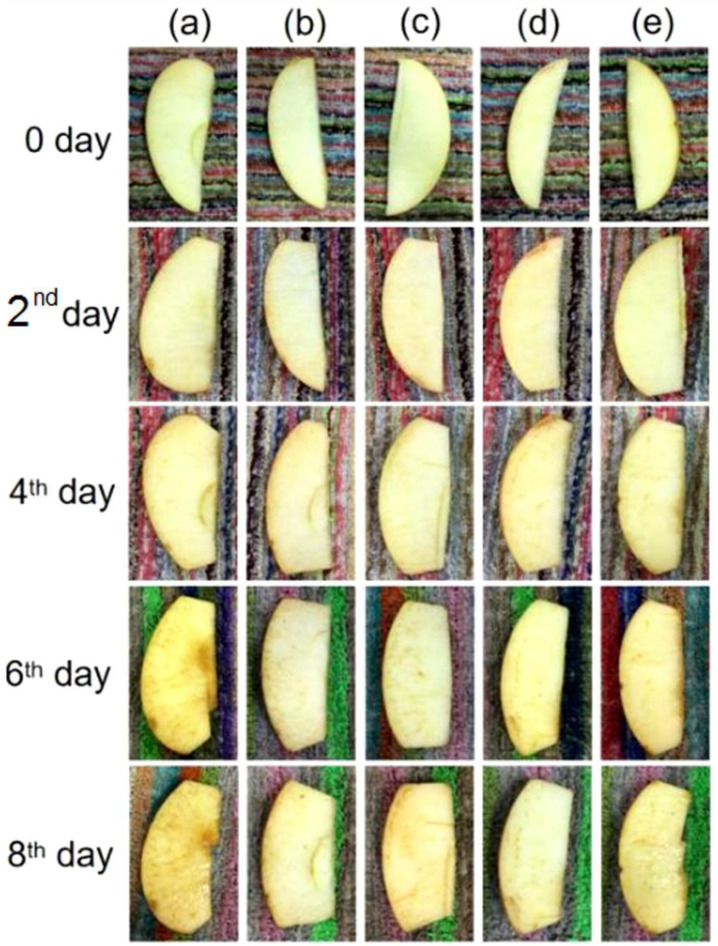
Ancillary packaging of apple slices and their appearance: (**a**) No fiber packaging; (**b**) F4 packaged group; (**c**) F5 packaged group; (**d**) F6 packaged group; (**e**) F7 packaged group.

As the direct contact packaging caused the fiber membrane to absorb water and collapse during the experiment, the apple slices were also packaged in secondary packaging to verify their long-lasting resistance to oxidation. The apple slices were packed at a temperature of 4 °C for a preset period of eight days without direct contact, and the changes in the process are shown in Figure 14 below. Groups (a) to (e) are the blank control and the apple slices packed with fibers F4 to F7, respectively.

Overall, oxidation was observed in the apple slices over time, while the changes were more pronounced in the unpacked blank control group. Groups (b) to (e), which were packed with fiber membranes, showed oxidation from day 6, indicating the presence of ferulic acid molecules that prevented the oxidation of the apple slices by free radicals by diffusion onto the food surface. The fiber membranes prepared in this experiment still had good antioxidant properties in the case of non-contact packaging.

A comparison of the apple slices after direct contact packaging and non-contact packaging showed that the fiber membranes in contact packaging were highly absorbent, and therefore, the apple slices showed a faster water loss. The faster water loss and the drying out of the apple slices might have been due to the small size of the apple slices. The difference between the preservation time of apple slices at 4 °C and at room temperature was significant, as the lowering of the temperature slowed down the oxidation process on the apple slices to a certain extent; on the other hand, the water loss from the apple slices was delayed. Both the lower temperature and the non-direct contact packaging helped to preserve the moisture in the apples.

### 3.13. The Browning Rates of Ancillary Fruit Packaging

**Figure 15:**
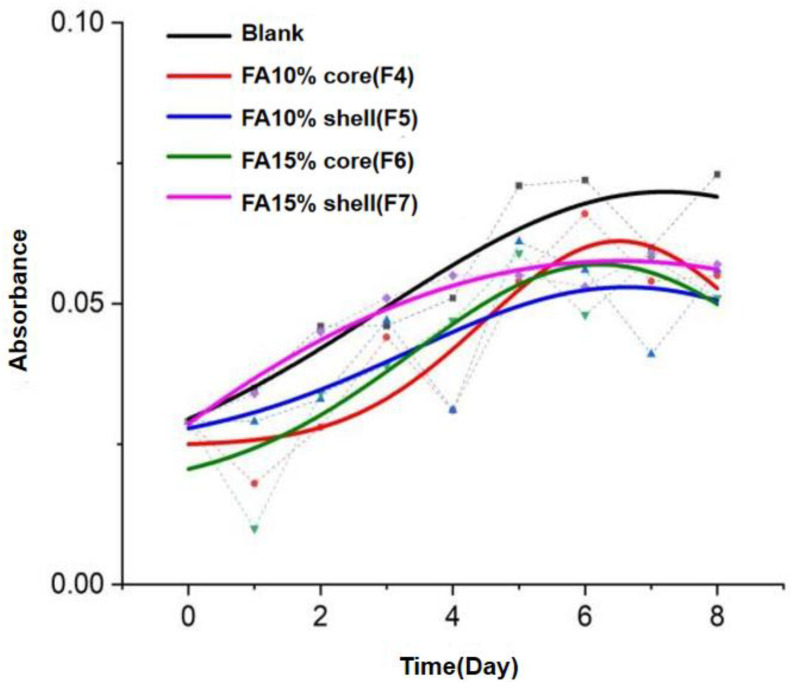
Qualitative analysis of the browning rate of apple slices after ancillary fruit packaging.

After the surface morphology of the apples had been observed, the amount of browning products was analyzed qualitatively by UV spectrophotometry in order to explore the degree of oxidation of the apple slices and the ability of the fiber membrane to retain freshness. The resulting browning rates of the apple slices are shown in Figure 15.

Due to the inconsistency of oxidation on the apple slices, the distribution of points after testing was not obvious. Therefore, the amount of browning product obtained was not consistently representative of the overall oxidation level when sampling the edges of the sliced apples. After the data obtained were fitted to a Gaussian equation, the fitted curves were found to be consistent with certain qualitative patterns and experimental expectations. The overall browning was higher in the blank control group, while the browning rate increased more slowly in all of the fiber-packed specimens. Of the four browning curves, the curves for F4 and F6 showed an increase followed by a decrease, possibly due to the heterogeneity in the degree of oxidation to which the apple slices were subjected. The heterogeneity may have been due to the fact that the core-loaded FA fibers released a lower amount of FA in the early stages and therefore did not provide good overall protection to all parts in the early stages. The increased release of FA over time resulted in the originally protected areas containing more antioxidant properties and therefore, a significant difference in the browning rate between the oxidized and unoxidized areas. In contrast, the curvature of the curves for the F5 and F7 fibers, which had loaded FA in the sheath layer, was less due to the timeliness of their FA release.

## 4. Conclusions and Perspectives

In this paper, a new core–sheath structure fiber of zein/PEO/FA was successfully prepared by coaxial electrospinning technology. The SEM and TEM results showed that the core–sheath structure of the fibers was smooth and straight in shape, with uniform diameter distribution. In addition, the component analysis confirmed that the internal structures of PEO, zein, and FA were compatible. The dissolution analysis showed that the core layer drug-loaded fibers had a continuous and stable release of FA, and the sheath layer drug-loaded fibers had a large initial release of FA. The fibers showed strong antioxidant activity within 24 h, with a maximum of 44.18% for F6 fibers loaded with 15% FA in the core layer. In the study of packed apple slices, fiber membrane packaging reduced the weight loss and browning rate of the apple slices and provided antioxidant preservation. The core-loaded fibers retained freshness for 10 h under contact packaging at room temperature and for six to eight days under non-contact packaging at 4 °C. Under contact packaging, the core-loaded fibers retained freshness for longer, while under non-contact packaging, the sheath-loaded fibers had a greater anti-browning capacity for the whole slices. Therefore, it could be used as an effective food antioxidant packaging material.

Both active ingredients and polymers that have natural sources are popular in a wide variety of biomedical applications, such as pharmaceutics and tissue engineering [80,81,82,83,84]. This is because of their greater essential bio-compatibility over synthetic polymers from chemical reactions [85,86,87]. In the drug delivery area, new excipients and techniques are continuously introduced by modern science and technology for developing novel drug delivery systems [88,89,90], particularly in the nano era [91,92]. Those strategies can be similarly exploited for developing novel food and fruit packaging materials for a better preserving effect. Based on natural macromolecules, the present study offered an example of strategies to achieve this. There will also be many new possibilities in the future.

## Figures and Tables

**Table 1:** Electrospinning processing parameters for the prepared fibers.

Fiber No.	Electrospinning	Voltage (kV)	Core Fluid			Sheath Fluid			Collection Distance (cm)
			Polymer	Functional Ingredient	Flow Rate (mL/h)	Polymer	Functional Ingredients	Flow Rate (mL/h)	
F1	Single-fluid	16.5	zein	-	0.2	-	-	-	10
F2	Single-fluid	6	PEO	-	0.6	-	-	-	15
F3	Coaxial	7.5	PEO	-	0.6	zein	-	0.3	20
F4	Coaxial	7.5	PEO	FA (10%)	0.6	zein	-	0.3	20
F5	Coaxial	7.5	PEO	-	0.6	zein	FA (10%)	0.3	20
F6	Coaxial	7.5	PEO	FA (15%)	0.6	zein	-	0.3	20
F7	Coaxial	7.5	PEO	-	0.6	zein	FA (15%)	0.3	20

## Data Availability

The data supporting the findings of this manuscript are available from the corresponding authors upon reasonable.
